# Comparative transcript profiling of gene expression between seedless Ponkan mandarin and its seedy wild type during floral organ development by suppression subtractive hybridization and cDNA microarray

**DOI:** 10.1186/1471-2164-13-397

**Published:** 2012-08-16

**Authors:** Wen-Ming Qiu, An-Dan Zhu, Yao Wang, Li-Jun Chai, Xiao-Xia Ge, Xiu-Xin Deng, Wen-Wu Guo

**Affiliations:** 1 Key Laboratory of Horticultural Plant Biology (Ministry of Education); National Key Laboratory of Crop Genetic Improvement, Huazhong Agricultural University, Wuhan, 430070, China

**Keywords:** Citrus, cDNA microarray, Differential transcript, Male sterility-like protein, Seedlessness

## Abstract

**Background:**

Seedlessness is an important agronomic trait for citrus, and male sterility (MS) is one main cause of seedless citrus fruit. However, the molecular mechanism of citrus seedlessness remained not well explored.

**Results:**

An integrative strategy combining suppression subtractive hybridization (SSH) library with cDNA microarray was employed to study the underlying mechanism of seedlessness of a Ponkan mandarin seedless mutant (*Citrus reticulata* Blanco). Screening with custom microarray, a total of 279 differentially expressed clones were identified, and 133 unigenes (43 contigs and 90 singletons) were obtained after sequencing. Gene Ontology (GO) distribution based on biological process suggested that the majority of differential genes are involved in metabolic process and respond to stimulus and regulation of biology process; based on molecular function they function as DNA/RNA binding or have catalytic activity and oxidoreductase activity. A gene encoding male sterility-like protein was highly up-regulated in the seedless mutant compared with the wild type, while several transcription factors (TFs) such as AP2/EREBP, MYB, WRKY, NAC and C2C2-GATA zinc-finger domain TFs were down-regulated.

**Conclusion:**

Our research highlighted some candidate pathways that participated in the citrus male gametophyte development and could be beneficial for seedless citrus breeding in the future.

## Background

Seedlessness is a desired fruit trait for consumers, and a fruit is considered to be seedless if it produces no seeds, traces of abortion seeds, or significant reduced-number of seeds [1]. Some plants can set seeds asexually through apomixis. However, in most flowering plants, seed initiation requires signals activated by the double fertilization event that occurs in the embryo sac, and seed is produced sexually from the fertilized ovule [2,3]. Various phytohormones such as gibberellins (GAs), auxins and cytokinins are involved in this signaling process [4-6]. GAs and jasmonic acid/jasmonate derivatives (JAs) were found to play crucial roles in plant reproductive development [7,8].

Citrus is one of the most important fruit crops with great economic and health value around the world [9]. However, some citrus varieties are seedy, and seedy fruits have constrained the development of fresh citrus market. Therefore, breeding seedless citrus varieties is a long-term pursuit for citrus breeders worldwide [10,11]. Nowadays, Satsuma mandarin and navel orange are two of the most famous and widely grown citrus varieties, mainly due to their seedless trait. For decades, great progress on seedless citrus breeding was made by traditional approaches such as sexual hybridization, seedling and bud sport mutation. However, due to the peculiarities of citrus reproductive biology such as long juvenile period and nucellar polyembryony, traditional breeding is inefficient and costly [12]. Modern biotechnological approaches (e.g. somatic hybridization) have potential to effectively expedite breeding process of citrus [13-15]. As most citrus varieties can produce fruits parthenocarpically [16], male or female sterility, embryo sac abortion, self-incompatibility, polyploidy and even environmental stress can result in seedless citrus fruits [17,18]. Actually there were some successful reports about seedless fruit production by genetic transformation. Ectopic expression of *iaaH* gene with *DefH9* as promoter to elevate auxin levels in placenta or ovules resulted in seedless fruits [19,20]. Another effective strategy was by specific expression of toxin proteins during early development of plant reproductive organs. Typical cases were the ectopic transformation of the *Barnase* gene from *Bacillus amyloliquefaciens*[21,22]. Potential cases were by specific expression of enzymes such as chloroplast Chaperonin 21 and ubiquitin extension protein S27a to induce cell disruption of seed tissues for parthenocarpic plants [11,23,24]. And in our laboratory, the *Arabidopsis thaliana MAC12.2* gene had been introduced into precocious trifoliate orange (*Poncirus trifoliata* [L.] Raf) for production of potential seedless fruits [25].

Male sterility (MS) is one of the main causes for seedless fruit production in citrus. In recent years, great progress on MS was made with annual plants especially rice [26,27], *Arabidopsis*[28] and oil-rape [29], and a serial of genes regulated tapetum, anther and pollen development were identified. However, there remained very limited information on MS of perennial woody plants such as citrus. Ponkan mandarin (*Citrus reticulata* Blanco) is a widely grown citrus variety in China. Within this variety, many variants were derived through sexual hybridization and mutation such as bud sport mutation. ‘Qianyang seedless’ Ponkan mandarin (QS) is an elite seedless variant selected from bud sport mutation of a common seedy Ponkan mandarin, and it can set fruits with no seeds (even no seed rudiments) in open orchard [30,31]. In this article, QS and a common seedy Ponkan mandarin ‘Egan NO.1’ (EG) were used for comparative study. These two mandarins shared highly close genetic relationship based on molecular marker analysis and showed no distinctly morphological differences except that QS was completely male sterile while Egan No 1 has normal flower. In order to gain general understanding on genes involved in this MS mutation, suppression subtractive hybridization (SSH) [32] combining with cDNA microarray was performed to detect differentially expressed genes. Several candidate genes and related pathways were focused in particular. Our research identified some useful genes which could be beneficial to citrus seedless breeding. The results could help to reveal the molecular mechanism of male sterility of Ponkan mandarin and shed light on seedless trait formation of other perennial woody plant at the gene expression level.

## Results

### Phenotype analysis of the floral organs of QS

**Figure 1:**
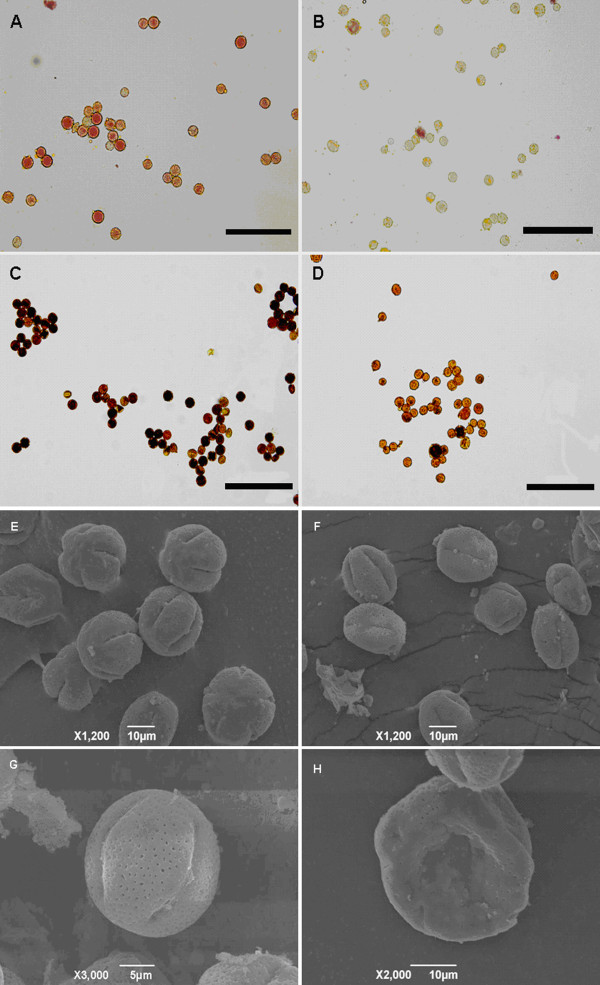
**Viability assay and SEM analysis of pollen grains. A, C, E, G** showed the pollen grains of EG; **B, D, F, H** showed the pollen grains of QS. Bar (**A, B, C, D**) = 100 μm.

Previous studies suggested that the floral organs (actually the whole plant) of QS had no morphological difference from the wild type. To further validate the phenotype of this seedless Ponkan mandarin, we measured the length of filament and pistil, and the average ratio of filament to pistil (filament length/pistil length) was 0.83 ± 0.01 for EG and 0.79 ± 0.01 for QS. And for EG, the pistil was 0.155 ± 0.01 cm longer than filament while for QS, the pistil was 0.166 ± 0.009 cm longer than filament. Above data further confirmed that the floral organs of both EG and QS had no morphological difference, and the seedless trait was not caused by malformation of reproductive organs. However, the number of pollen grains per anther of QS was 9.5% less than that of EG. The pollen dying viability of QS was 6.0% ± 1.0% (or 6.5% ± 1.0% for I_2_-KI_2_ staining) in striking contrast to the high viability of 93.8% ± 0.9% (or 89.6% ± 2.5% for I_2_-KI_2_ staining) for EG. Pollen germination test found that no pollen of QS could germinate. Furthermore, SEM assays showed abnormal structures of the pollen grains of QS (Figure 1), confirming that QS is male sterile.

### Construction of SSH-cDNA libraries and overall feature of the differential transcript profiling

To identify genes associated with the MS of QS, SSH cDNA libraries (both forward and reverse) were constructed from floral organs of QS and EG. A total of 6,048 cDNA clones derived from the SSH-cDNA libraries including 4,195 from the forward library and 1,853 from the reverse one were successfully amplified, and then used for a custom cDNA microarray. Each cDNA clone has triplicate spots on the array. The RNA samples of the four developmental stages (SF, MF, BF and OV) were used for array-hybridization. The fluorescent dye-labelled cDNA and hybridization strategy was employed for the microarray assay.

**Figure 2:**
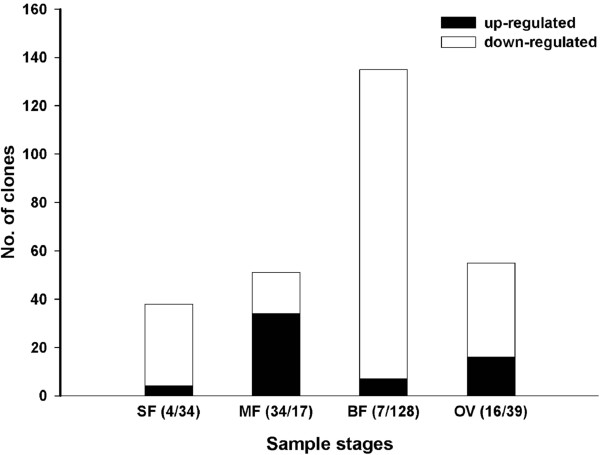
**Number of clones significantly up- and down-regulated in QS during four developmental stages.** Each two numbers in parentheses of x-axis indicated the numbers of clone up- and down-regulated respectively.

From the 6,048 clones printed on the glass slide, 279 cDNA clones (278 non-redundant) were differentially expressed (false discovery rate (FDR) <0.05 and a fold change ≥ 2) between QS and EG. Among these cDNA clones, 218 (78%) were down-regulated while only 61 (22%) showed up-regulated expression across the four developmental stages; and the differentially expressed clones peaked at full bloom stage (BF) (Figure 2). At this stage, many more clones showed down-regulated than up-regulated expression. During the four developmental stages, one clone (GenBank accession no. JU497336) encoding a putative cysteine protease (tr[B9RRA4]) showed down-regulated expression at BF stage but up-regulated at OV stage (young ovaries of 2–3 days after flowering).

### Sequencing of the differentially expressed clones and EST analysis

Among the 279 differentially expressed clones, 255 non-redundant clones were subjected to one single-pass sequencing. In all, 237 high-quality ESTs (average length was 496 bp) were yielded after eliminating vectors and unreliable sequences. These ESTs were assembled using CAP3 program, and 133 unigenes (43 contigs and 90 singletons) were obtained with sequence redundancy of 43.9%. The majority of the contigs (38) contained 2–5 ESTs, whereas only 5 contigs contained 6–11 ESTs, indicating an ideal normalization and subtraction. Of the 133 unigenes, 80 (60.1%) showed differential expression at BF stage. Subsequently, BLASTX search of the UniProt database showed that 20 unigens (15.0%) did not have significant hits (E-value ≤1.0 × e^-5^). However, when the 20 unigenes were used in BLASTN (E-value ≤1.0 × e^-10^) search of the *Citrus clementina* transcript database [33,34] with local Blast software ( ftp://ftp.ncbi.nlm.nih.gov/blast/executables/release/LATEST/), 17 genes had significant hits and high scoring pairs (HSP) showed high nucleotide identity. It suggested that these 20 unigenes were unique for citrus, and three of them were novel citrus genes.

**Figure 3:**
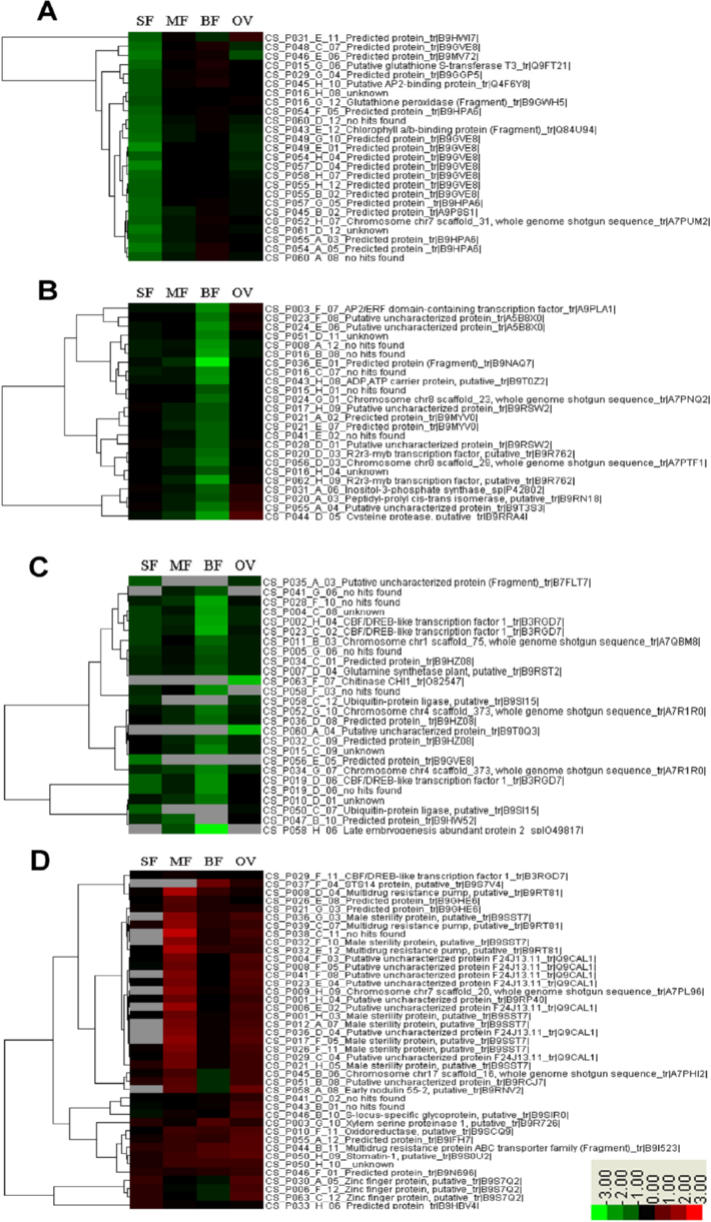
**Cluster analysis of expression profiles of altered expressed genes in the QS versus EG.A** showed a cluster of ESTs that were down-regulated mainly at squaring stage (SF) when the tetrads were produced and the microsporocyte underwent meiosis. **B** showed ESTs that were down-regulated especially at full bloom stage (BF). **C** and **D** suggested a cluster of ESTs that were down-regulated and up-regulated constitutively during the four developmental stages respectively. The ratio value was log2 transformed for each gene and used for the hierarchical clustering analysis.

Based on the microarray analysis, the relative expression profiles of all 255 ESTs were performed hierarchical clustering with cluster software (version 3.0). Four typical relative expression patterns were observed in QS versus EG at four developmental stages. Figure 3A and 3B showed a group of clones down-regulated mainly at squaring stage (SF) and full bloom stage (BF), respectively, while the other two groups of clones were down/up-regulated constitutively during the developmental stages (Figure 3C and 3D). In addition, candidate genes with putative function that could be important for the MS of QS were specifically collected (Table 1). It is noteworthy that 27.7% of the unigenes (not listed in the table) were only annotated as putative proteins or with no defined biological process besides 15% unigenes with no hits in the database.

**Table 1:** List of selected candidate functional genes related to the formation of the phenotype of QS

**GenBank accsession no.**	**EMI**^**a**^	**Description**^**b**^	**e-Value**^**c**^	**Clones**^**d**^
**Up-regulated**				
JU497309	B9SST7	Male sterility protein	2E-77	7
JU497311	B9S7V4	STS14 protein	6E-23	3
JU497315	Q5CD81	(E)-beta-ocimene synthase	1E-120	2
JU497324	B9RXQ0	Tryptophan synthase beta chain	6E-91	2
JU497327	B9RT81	Multidrug resistance pump	5E-50	3
JU497333	B9S7Q2	Zinc finger protein	1E-67	3
JU497348	A9ZN18	Geranyl-diphosphate synthase	3E-11	5
JU497417	B9R726	Xylem serine proteinase 1	7E-24	1
JU497359	B9SCQ9	Oxidoreductase	2E-36	1
JU497418	A1ECJ7	Putative miraculin-like protein 2	7E-24	1
JU497422	A9XCN2	Putative DNA binding protein	2E-24	1
JU497389	B9I523	Multidrug resistance protein ABC transporter family	1E-121	2
JU497397	B9SIR0	S-locus-specific glycoprotein	2E-45	1
JU497403	B9S0U2	Stomatin-1, putative	1E-07	1
**Down-regulated**				
JU497308	B9S7Q1	Zinc finger protein	1E-140	2
JU497318	B9S1E9	Transcription factor AtMYC2	4E-20	2
JU497321	B2VQE0	Methionine synthase	1E-107	4
JU497323	P42802	Inositol-3-phosphate synthase	1E-112	4
JU497331	B9SR02	Multicopper oxidase	6E-78	2
JU497332	B3RGD7	CBF/DREB-like transcription factor	6E-44	4
JU497336	B9RRA4	Cysteine protease	2E-25	2
JU497338	Q8VWL8	Beta-mannosidase	6E-64	2
JU497342	O82547	Chitinase CHI1	8E-28	7
JU497343	O49817	Late embryogenesis abundant protein	2E-22	2
JU497344	B9SI15	Ubiquitin-protein ligase	2E-54	2
JU497351	A9PLA1	AP2/ERF domain-containing transcription factor	2E-46	1
JU497352	B9T724	GATA transcription factor	4E-62	1
JU497353	B9RZK6	Protein COBRA	4E-82	1
JU497354	A2IB54	Mitogen-activated protein kinase	4E-85	1
JU497356	B9RST2	Glutamine synthetase plant	3E-71	1
JU497357	Q3KN68	Isoflavone reductase-like protein 5	2E-56	1
JU497361	B9SQM6	Transcription factor	3E-34	1
JU497362	Q9FT21	Putative glutathione S-transferase T3	2E-07	1
JU497364	B9GWH5	Glutathione peroxidase (Fragment)	2E-25	1
JU497367	B9RN18	Peptidyl-prolyl cis-trans isomerase	3E-54	1
JU497368	B9R762	R2R3-myb transcription factor	1E-69	1
JU497369	O82547	Chitinase CHI1	8E-28	1
JU497419	P83948	Pectinesterase-3	1E-46	1
JU497372	B9NBQ9	AP2/ERF domain-containing transcription factor	1E-108	1
JU497373	Q9ZRC9	ACC oxidase	6E-36	1
JU497421	A5YWA9	NAC domain protein	1E-51	1
JU497378	Q7Y066	Plasma membrane H^+^-ATPase	1E-114	1
JU497423	B9RIP3	Hevamine-A	1E-22	1
JU497424	B9T0Z2	ADP/ATP carrier protein	1E-06	1
JU497427	Q84U94	Chlorophyll a/b-binding protein (Fragment)	2E-14	1
JU497388	A7XUL4	dehydration-responsive element binding protein	1E-14	1
JU497391	Q4F6Y8	Putative AP2-binding protein	6E-15	1
				
JU497398	Q6EV47	Non-specific lipid-transfer protein (Fragment)	1E-45	1
JU497401	B9SJL5	Amine oxidase	1E-57	1
JU497405	B9S925	Zinc finger protein	3E-88	1
JU497406	B9T868	Putative peroxidase C3 (Fragment)	8E-26	1
JU497434	Q8H2A1	Caffeoyl CoA O-methyltransferase (Fragment)	3E-11	1
JU497412	B9HGW6	Glutaredoxin	6E-20	1
JU497413	B9R762	R2R3-myb transcription factor	3E-21	1

^a^ The EMI codes of the most similar genes to the EST sequences.

^b^ The description of sequences based on Uniprot database.

^c^ The best e-value from a BLASTx search for corresponding EST sequences.

^d^ The number of sequenced clones in the libraries.

**Figure 4:**
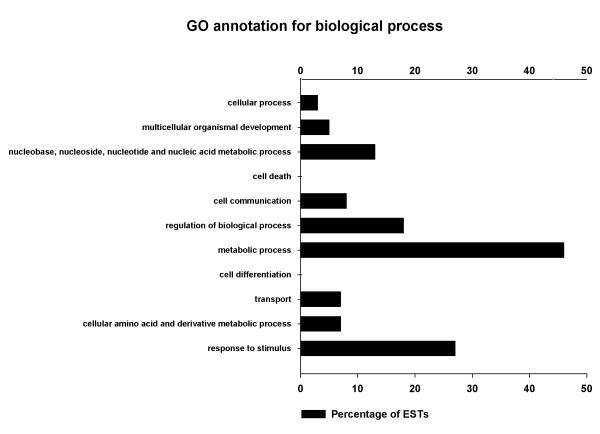
**Distribution of the unigenes according to the biological process (2nd level GO terms).** A total of 86 unigenes were annotated. As one gene product could be assigned to more than one GO term, the percentages will add up to more than 100%.

GO annotations were conducted and three categories representing molecular functions, biological processes, and cellular components were assigned. Figure 4 showed the percentage distributions of GO terms (2nd level GO terms) based on biological process. It indicated that during the floral organ development, the majority of differentially expressed genes were involved in metabolic process (46%) or responded to stimulus (27%) and regulation of biological process (18%). In addition, the other two GO categories (molecular functions and cellular components) were also generated (data not shown). In the molecular function category, large proportion of unigenes may have binding activity (59%), catalytic activity (19%), or oxidoreductase activity (11%), while the cellular components consisted mainly of intracellular (57%) and membrane (23%).

### Metabolic pathways involved in formation of seedless fruit

**Figure 5:**
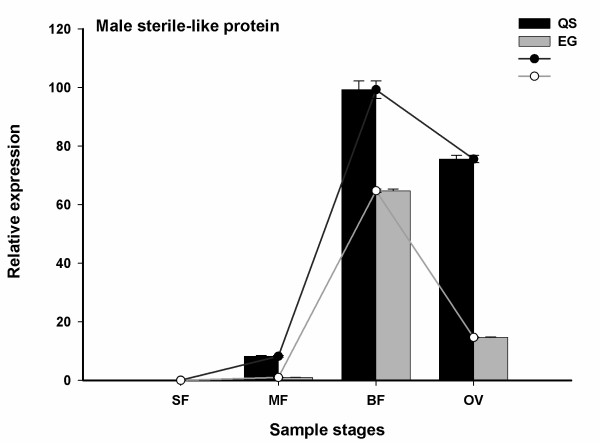
**Relative expression (y-axis) of male sterile-like protein in QS versus EG during four developmental stages (x-axis) by qRT-PCR.** Columns and bars represent the means and standard errors (*n* = 3) respectively.

As large proportion of altered expressed genes were involved in varieties of metabolic processes. Based on the KEGG (Kyoto Encyclopedia of Genes and Genomes) analysis, 36 different metabolic pathways were altered during the four developmental stages. Among these pathways, nine (25%) were related to amino acid metabolic pathway (Table 2), and genes involved in carbohydrate and energy metabolism showed down-regulated expression during subsequent developmental stages of floral organs. Besides, genes related to specific secondary metabolism such as terpenoids and polyketides metabolism were also found to be altered. Interestingly, a gene (JU497309) encoding fatty acyl-CoA reductase, which may be involved in lipid metabolic process, was identified (Table 1). This gene was found highly homologous with putative male sterile protein (GI: 255576327) in castor bean, fatty acyl-CoA reductase 3 (GI: 359500474) in poplar and male sterile 2-like protein (*MS2*) (GI: 3549681) in *Arabidopsis*. Herein, this gene was named as male sterile-like protein. And qRT-PCR analysis showed its expression level increased from SF to BF stages and then declined at OV stage. The expression pattern was similar in both QS and EG; however, it showed obviously higher expression level in QS than in EG during the developmental process (Figure 5).

**Table 2:** List of differentially expressed genes involved in amino acid, carbohydrate, energy, terpenoid and polyketides metabolism based on KEGG pathway database

**KEGG pathways**	**EC number**	**Putative function**	**QS/EG**			
			**SF**	**MF**	**BF**	**OV**
**Amino acid metabolism**						
JU497356	6.3.1.2	Glutamate-ammonialigase	0.71±0.03	0.84±0.05	**0.50**±0.01	0.79±0.03
JU497374	6.3.5.4	Asparagine synthase	**0.50**±0.03	1.15±0.08	1.20±0.09	1.02±0.06
JU497373	1.14.17.4	Aminocyclopropane carboxylate oxidase	0.90±0.02	1.52±0.05	**0.52**±0.03	1.15±0.09
JU497321	2.1.1.14	5-methyltetrahydropteroyltriglutamate	1.62±0.11	0.97±0.08	**0.50**±0.02	0.90±0.08
		-homocysteine-methyltransferase				
JU497330	4.2.1.78	(S)-norcoclaurine synthase	1.04±0.11	**1.94**±0.28	1.29±0.04	1.17±0.13
JU497338	3.2.1.21	Beta-glucosidase	0.94±0.08	**0.47**±0.04	**0.18**±0.01	0.88±0.17
JU497364	1.11.1.1	Phospholipid-hydroperoxide	**0.53**±0.04	0.97±0.10	0.97±0.05	1.08±0.01
		-glutathione peroxidas				
JU497324	4.2.1.20	Tryptophan synthase	1.86±0.13	**2.64**±0.16	1.03±0.05	0.70±0.05
JU497377	2.4.1.12	Indole-3-acetatebeta-glucosyl transferase	**0.51±0.01**	1.51±0.04	1.25±0.02	**0.48**±0.04
**Carbohydrate metabolism**						
JU497385	3.2.1.14	Chitinase	1.15±0.15	1.28±0.05	1.00±0.17	**0.45**±0.02
JU497313	1.13.99.1	Inositol oxygenase	1.04±0.09	1.08±0.04	0.79±0.07	**0.49**±0.03
JU497323	5.5.1.4	Inositol-3-phosphate synthase	1.11±0.08	1.17±0.20	**0.48**±0.03	1.42±0.13
JU497357	1.3.1.45	2{prime}-hydroxy isoflavone reductase	1.10±0.05	1.28±0.07	**0.44**±0.01	1.17±0.04
**Energy metabolism**						
JU497406	1.11.1.7	Peroxidase	0.99±0.05	1.02±0.01	1.12±0.09	**0.54**±0.06
JU497378	3.6.3.6	Proton-exporting ATPase	0.99±0.05	0.90±0.02	**0.51**±0.02	0.94±0.03
**Terpenoids and polyketides metabolism**						
JU497376	1.1.1.295	Momilactone-Asynthase	1.42±0.54	1.09±0.20	1.05±0.03	1.10±0.10
JU497315	4.2.3.15	Myrcene synthase	1.24±0.04	**2.28**±0.16	0.52±0.01	1.10±0.11
JU497325	4.2.3.20	(R)-limonene synthase	1.27±0.18	0.91±0.03	**0.50**±0.02	1.10±0.17

### Differential expression of transcription factor genes

**Figure 6:**
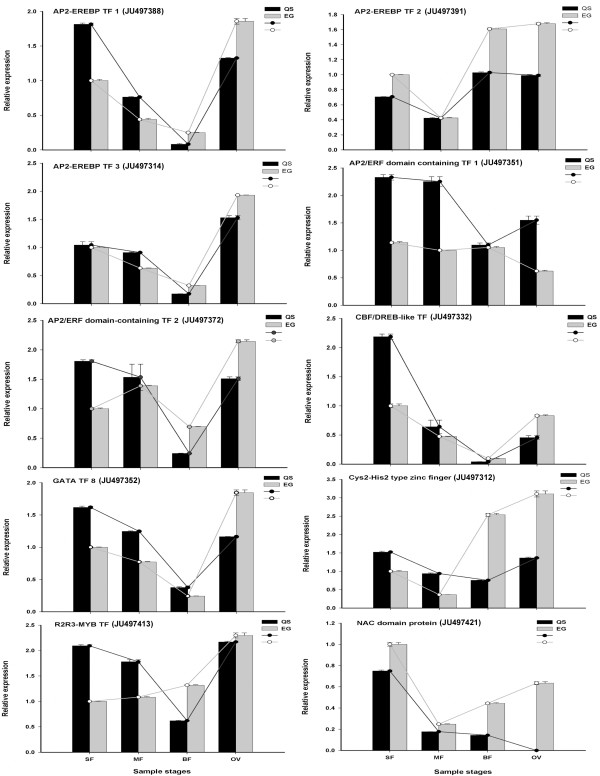
**Relative expression pattern of six AP2-ERF family TFs, two zinc-finger TFs, one MYB family TF and one NAC domain TF.** The accession number of each TF was given inside the parenthesis. Relative expression was defined as the expression level in QS versus EG. Columns and bars represent the means and standard errors (*n* = 3) respectively.

It is noteworthy that among the 133 unigenes, 12 were assigned to the category of transcription factor (TF) based on plant TF database ( http://planttfdb.cbi.edu.cn/). Figure 6 showed the specific expression pattern of six AP2-ERF family TFs, two zinc-finger TFs, one MYB TF and one NAC TF using qRT-PCR assay. These TFs (except of NAC TF) had similar expression profile during the four developmental stages between EG and QS. For instance, among six AP2-ERF TFs, four (AP2-EREBP TF1, AP2-EREBP TF3, AP2/ERF domain containing TF2 and CBF/DREB-like TF) showed co-expression pattern like “V” type. It showed that the gene expression level in QS was higher than that in EG from SF stage to MF stage; however, these genes were subsequently repressed more obviously in QS from MF stage to BF stage, and the gene expression level was down-regulated mostly at BF stage. Two zinc-finger TFs (GATA TF8 and Cys2-His2 type) and one R2R3-MYB TF likewise showed similar “V” type-variation tendency. The other two AP2-ERF TFs (AP2-EREBP TF2 and AP2/ERF domain containing TF1) showed “V”-like type expression pattern in QS. However, the expression pattern of AP2/ERF domain containing TF1 was somehow different from others, as it showed relatively stabilized expression level during the four stages in EG. As for NAC TF, its expression level was down-regulated obviously at BF and OV stages in QS compare with EG. It was notable that no expression was observed at OV stage in QS. The results suggested that these TFs could play important roles in the seedless phenotype formation, and the relative expression level in QS versus EG seemed to be key factor in this process.

### Verification of microarray data

Two approaches were used to examine the quality of the microarray data. First, as one contig was assembled by several ESTs that were arrayed at random location in the microarray, so these ESTs sharing similar sequence or encoding the same gene would share similar expression pattern. Additional file 1: Figure S1 showed that four ESTs (F2-13 G, F6-15I, F7-18O, F8-12A) were assembled into one unigene (JU497321) which encoded methionine synthase, and these four ESTs truly shared similar expression pattern. For the other approach, qRT-PCR was performed on 11 unigenes using gene-specific primer pairs. Expression patterns were compared at the four developmental stages between QS and EG. Additional file 2: Figure S2 showed the correlation analysis of the ratio values of differential expression level from microarray to that from qRT-PCR. Linear regression [(average microarray ratio value) = *a* (qRT-PCR value) + *b*] analysis showed a good coefficient of variation (*R*^*2*^ = 0.847). These results confirmed the reliability of the microarray data.

## Discussion

Here, we combined SSH and microarray techniques to investigate potential mechanism underlying seedlessness in Ponkan mandarin. SSH was proved to be an efficient and popular approach to enrich and identify differentially expressed genes between wild-type and its mutant or treatment [35,36]. However, because of high sensitivity of SSH, usually a large number of clones could be obtained but inevitably included some false-positive ones. Screening the SSH libraries to identify some candidate genes using microarray and to validate using qRT-PCR has proved to be a high-throughput and efficient way [37-39]. However, relatively few clones were isolated in this study. Of the 6,000 clones, only 279 cDNA clones were identified as differentially expressed. Such results may suggest that there were little variations between QS and EG mandarins in gene expression. It was hypothesized that bud sport mutant was likely caused by single gene mutation, DNA methylation or retroelement activity [40,41]. In this research, various types of DNA markers including SCAR [42], and SSR (172 pairs of primers), MSAP (96 pairs of primers) and AFLP (13 pairs of primers) were employed to analyze the polymorphism between these two mandarins, and no repeatable polymorphic bands were detected (data no shown). These results suggested that very few nuclear genes were altered during the developmental stages.

For the four developmental stages we chose, immense efforts were taken to determine which time-point was pivotal for stamen development, but there has no criteria for citrus gametophyte development. Though criteria for gametophyte development was available in model plant *Arabidopsis*[43], it can not be directly applied herein. Semi-thin and paraffin sections were performed in this study to survey the microsporogenesis of QS, and it was found that abnormal tetrads produced at the tetrad stage and subsequently the microsporocyte underwent abnormal meiosis. This process mainly occurred at SF stage (the diameter of floral organs is about 3 mm) (unpublished data). Additionally, large proportion (about 59.7%) of differentially expressed genes was found in BF when the anthers and pollen grains were almost mature, indicating that this time-point might be also important.

### Amino acid metabolic process

Of the metabolic pathways with altered expressed genes, 25% were involved in amino acid metabolism. Amino acids were not only primary metabolic products for normal growth and development but also cell signaling molecules and regulators of gene expression and protein phosphorylation cascade [44]. Interestingly, among these amino acid metabolism pathways, two genes were down-regulated across the developmental stages in QS versus EG, one (JU497356) encoding glutamate-ammonialigase (EC 6.3.1.2), the other (JU497338) encoding beta-glucosidase (EC 3.2.1.21). In higher plants, glutamate-ammonialigase catalyzes ATP-dependent conversion of glutamate and ammonia into glutamine which occupies a central position of amino acid metabolic pathway [45], and this metabolic process is critical for coordinating metabolic balance in rice [46]. And beta-glucosidase could be used for the cellulosic ethanol industry [47] and has diversity of functions in plants. In maize, *Zm-p60.1* encoding a beta-glucosidase could release active cytokinin, and might function *in vivo* to supply the developing maize embryo [48]. Additionally, some beta-glucosidases affect the properties of cell wall [49] and are associated with freezing tolerance, such as the *SFR2* in *Arabidopsis*[50]. Some beta-glucosidases are related to the efficiency of microspore embryogenesis [51]. It is noteworthy that a gene (JU497374) encoding asparagine synthase (EC 6.3.5.4) was down-regulated exclusively at SF (early stage of stamen development). And asparagine is one central intermediate in nitrogen assimilation and transportation in plant [52,53]. Recent studies showed that this gene played important role in defense against pathogens and salt stress [54,55]. Additionally, genes related to carbohydrate metabolism and energy metabolism also showed down-regulated expression in QS mainly at BF and OV (late stage of stamen development). These results suggested that the vital activities of QS weakened during early development stages of stamen, and the metabolic process of nutrition and energy was also impaired at subsequent stages of stamen development especially when the stamen was mature.

Two genes involved in cysteine/methionine metabolism and participated in the biosynthesis of ethylene were also identified in this study. One (JU497321) encodes 5-methyltetrahydropteroyltriglutamate-homocysteine S-methyltransferase (EC 2.1.1.14) is likely involved in the biosynthesis of L-methionine. And the methionine can be transformed into S-adenosylmethionine (SAM) (the precursor of ethylene) [56]. The other one (JU497373) encodes aminocyclopropane carboxylate oxidase (EC 1.14.17.4) and is a pivotal enzyme during the biosynthesis of ethylene. In addition, genes involved in the synthesis of IAA (indole-3-acetic acid) were also identified such as a gene (JU497377) encoding Indole-3-acetatebeta-glucosyltransferase (EC 2.4.1.121). These results implied that the endogenous phytohormones might be involved in the male gametophyte development of citrus.

### Transcription factors

It was known that floral organ formation and function were influenced by TFs regulation. In our research, twelve unigenes were assigned to the category of transcription factor, and six of them were identified as AP2-ERF family members. AP2-ERF TF containing highly conserved AP2/ERF DNA-binding domain, is a large family unique in plant. In our research, four AP2-ERF members showed similar expression pattern. AP2-EREBP TF1 was closely homologous with atERF107 (AT1G19210). This gene was likely involved in the regulation of gene expression by stress factors and by components of stress signal transduction pathways. However, until now, no experimental evidence was available. AP2-EREBP TF3 showed high similarity with ERF5 (AT5G47230.1). ERF5 might play an important role in plant innate immunity likely through coordinating chitin and other defense pathways [57]. Other research suggested that ERF5 and ERF6 might potentially overlap in their function and acted as positive regulators of JA/ethylene-mediated defense [58]. In tomato, this gene was mainly involved in responses to drought and salt stresses [59]. As for AP2/ERF domain containing TF2, its closest relative was ERF104 (AT5G61600.1). Recent studies showed that ERF104 was *in vivo* substrate of MPK6, and ethylene could release ERF104 and allow liberated ERF104 to access target genes related to plant defense [60]. CBF/DREB-like TF was of high similarity with CBF4 (AT5G51990.1) which was critical regulator involved in cold acclimation and drought adaptation [61,62].

In addition, AP2-EREBP TF2 was highly homologous with RAP2.4 (AT1G78080.1). RAP2.4 acted at or downstream of a converging point of light and ethylene signaling pathways, and it coordinately regulated multiple developmental processes and stress responses [63]. As for AP2-ERF domain containing TF1, its expression pattern was different from other five members. It showed high similarity with DREB26 (AT1G21910.1). In plant, RAP2.6, RAP2.6 L, DREB26 and DREB19 exhibited tissue specific expression and participated developmental processes as well as biotic and/or abiotic stress signaling [64]. Though previous researches emphasized the functions of these AP2-ERF TFs on resistance against biotic and abiotic stresses, AP2-ERF TFs were also participated in plant development such as embryo patterning [65], and stamen emergence [66].

Additionally, two MYB (R2R3-MYB) transcription factors also showed differential expression between QS and EG. In plant, MYB TF family was categorized into 3 subfamilies according to the number of adjacent repeats of MYB-domain. Of them, R2R3-MYB subfamily contains the largest number of members. Like the AP2-ERF TF family proteins, MYB family proteins also function in various plant-specific processes. In *Arabidopsis*, MYB TFs were found as key regulators involved in development, metabolism and biotic and abiotic stress responses. Among these MYB TFs of *Arabidopsis*, AtMYB26 is involved in determining endothecial cell development within the anther and is essential for anther dehiscence [67]. AtMYB33 and AtMYB65 redundantly facilitate anther and pollen development [68]. AtMYB80 regulates exine formation and acts downstream of AtMYB35; and AtMYB103 is required for tapetal development and microsporogenesis, especially for callose dissolution and exine formation [69,70]. AtMYB125 positively control male germ cell division and commit progenitor germ cells to sperm cell differentiation [71,72]. In rice, *CSA* gene encoding MYB TF functions as a key transcriptional regulator for sugar partitioning during male reproductive development, and the *CSA* mutant showed reduced levels of sugars and starch in floral organs which lead to MS.

Interestingly, in our results, one MYB TF showed similar expression pattern with AP2-ERF TFs that down-regulated at BF stage when the anther and pollen grains are mature. This MYB TF termed as R2R3-MYB TF was closely related to ATMYBR1/ATMYB44 (AT5G67300.1), and AtMYB44 was likely to enhance drought and salt stress tolerance by suppressing the expression of genes encoding PP2Cs, which was described as negative regulators of ABA signaling [73]. Previous report showed that AtMYB44 was with changed expression during late embryogenesis and seed maturation [74]. And notably there was a NAC domain protein (JU497421) highly homologous with ANAC102 (AT5G63790.1). ANAC102 was an important regulator of seed germination and activated a seed-specific subset of genes under low-oxygen stress; it was also necessary for the viability of *Arabidopsis* seeds following low-oxygen treatment [75].

In summary, these results suggested that these AP2-ERF TFs and the MYB TF functioned redundantly and coordinated with other TFs which involved in the complex network regulating floral organ development. Further research should emphasize on the isolation of proteins interacted with these TFs.

## Conclusion

An integrative approach combining SSH and microarray was employed to explore the transcriptional changes of a seedless bud sport mutant of Ponkan mandarin. A number of differentially expressed genes were identified. And the majority of genes were down-regulated in the mutant, especially those related to basic metabolic process. Metabolism of nutrition and energy might be impaired during male gametophyte development of the mutant, and TFs and phytohormones might play important regulatory roles during this process. Our research gained general information of citrus MS at transcription level and could provide some clues for further exploration of MS in citrus species.

## Methods

### Accession numbers of sequences and microarray data

All the sequences generated in the study were deposited in GenBank with accession numbers from JU497308 to JU497435. Five sequences which are shorter than 200 bp longer than 100 bp are attached in Additional file 3.

Microarray data and experimental information from this study were deposited in the Gene Expression Omnibus ( http://www.ncbi.nlm.nih.gov/geo/) under accession number GSE38094.

### Plant materials and phenotype analyses

**Figure 7:**
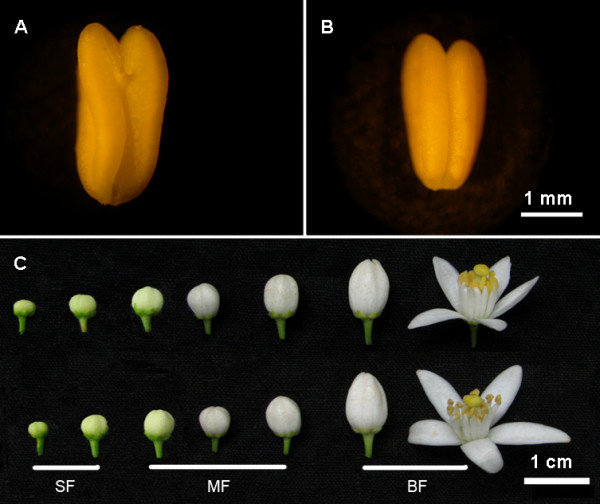
**Flower organs at different developmental stages and mature anthers.A**, **B** showed the anthers of EG and QS respectively; **C** showed the flower organs including SF, MF, BF stages, the upper row was EG.

Two Ponkan mandarin (*Citrus reticulata* Blanco) cultivars, Qianyang seedless (QS, mutant type) and Egan NO.1 (EG, a common seedy Ponkan mandarin, wild type) were grown in the same orchard of ‘Fenghuangshan’ citrus production area in the city of Dangyang, Hubei province, China. These two scion cultivars were seven years old when sampling in 2010, with trifoliate orange (*Poncirus trifoliata* L. Raf.) as the rootstock. Flower samples were collected from both cultivars in parallel including 4 continuous phonologically developmental stages (Figure 7C): squaring stage (SF, about 20 DBF), medium bud stage (MF, about 10 DBF), flowers at full bloom stage (BF) and young ovaries of 2–3 days after flowering (OV). All the flowers were bagged to prevent cross-pollination, and when sampled in the field, all the samples were frozen in liquid nitrogen as quickly as possible and then stored at −80°C until needed.

The morphology of mature anthers were investigated with fluorescence stereo-microscope (Figure 7A; 7B) (Leica MZ FLIII, German) and image was captured with a digital camera (Nikon Coolpix, Japan). The pollen grain number per anther was counted. In brief, anthers from mature flowers were collected and mixed randomly, each time 40 anthers were dissected and pollen grains were suspended in 25 mL sterile water with 4–5 drops of surfactant (Tween-20, Amresco solon, OH). The viability of mature pollen grains were evaluated by dying with 1% acetic acid magenta as well as 1% iodine potassium iodide (I_2_-KI_2_) solution. After staining for 5 min, pollen grains were observed using BX-61 fluorescence microscope (Olympus, Japan) and Images were captured with DP70 CCD digital camera system. At least 1,000 pollen grains were counted. These experiments were repeated three times. The morphology of pollen grains was examined by scanning electron microscope (SEM) (NTC JSM-6390LV, Japan). For SEM, anthers at various developmental stages were pre-fixed with 2.5% glutaraldehyde in 0.1 M sodium phosphate buffer (pH 7.2) for 24 h, dehydrated twice using a gradient ethanol serial (30%-50%-70%-85%-95%-100%), then replaced ethanol with isopentyl acetate for 20 min. After that, samples were dried with critical-point drying method then sputtered coating with gold. Representative images were captured.

### RNA extraction and mRNA isolation

The materials (floral organs) for RNA extraction were sampled from at least six independent plants, and mixed randomly. Total RNA from flower samples at four stages (SF, MF, BF and OV) were extracted with modified Trizol method according to [76]. The RNA pellets were washed with 75% (V/V) ethanol twice, dissolved in RNase-free water and stored at −80°C until use. By mixing equal amount of RNA of the four stages, RNA pools from both QS and EG were established in parallel. Then mRNA was isolated from each of the RNA pools using the Oligotex mRNA mini kit (Qiagen, Germany). The quality of RNA was determined by Nanodrop 1000 spectrophotometer (*Thermo Scientific*, Wilmington, DE, USA) and 1.2% agarose gel electrophoresis.

### Suppression subtractive hybridization (SSH) cDNA libraries construction and cDNA inserts amplification

Two micrograms of mRNA was used to synthesize cDNA for suppression subtractive hybridization (SSH). The SSH was performed with the PCR-select^TM^ cDNA subtraction kit (Clontech, Palo Alto, CA, USA) according to the user manual. And both forward (the seedless cultivar QS as tester and the seedy cultivar EG as driver) and reverse (EG as tester while QS as driver) SSH were conducted. For cDNA libraries construction, two hybridizations were performed followed by two rounds of PCR amplifications to enrich the desired differentially expressed sequences. Then the second PCR-amplified cDNAs were purified and ligated into the T/A cloning vector pMD18-T (Takara, Japan) overnight at 4°C. Then the ligated products were transformed into Electro MAX^TM^ DH5α-E^TM^ cells (Invitrogen, USA) and incubated at 37°C, 160 r/m for 1 h, then cultured on SOB-MgCl_2_ solid media with ampicillin (60 μg ml^-1^) to generate the primary cDNA libraries. The transformed white bacteria were randomly picked and grown on 384-well plates containing Luria Broth (LB) liquid media with ampicillin (100 μg ml^-1^) at 37°C overnight (about 16 h). Glycerol (Amresco, USA) (4.4% final) was added for storage at −80°C.

A total of 8,000 cDNA clones were randomly picked from forward and reverse SSH libraries and used as for subsequent PCR templates. Each PCR was performed in a 100 μl reaction mixture using nested primers of SSH according to [77]. The PCR products were precipitated with equal amount of isopropyl alcohol and washed with 75% (V/V) ethanol, then re-suspended in 40 μl sterile water. The yield and quality of the PCR products were determined by Nanodrop 1000 spectrophotometer (*Thermo Scientific*, Wilmington, DE, USA), and then run on 1.2% agarose gel and examined by Bio-Rad UV spectroscopy (Bio-Rad Laboratories, Washington, DC, USA) to confirm single clone (Additional file 4: Figure S3). Finally the validated PCR products were stored at −80°C for custom microarray.

### Microarray slides fabrication and preparation of fluorescent dye-labelled cDNA

About 40 microlitre of PCR products were re-precipitated by adding 100 μl of anhydrous ethanol and were dissolved in EasyArray^TM^ spotting solution (CapitalBio Corp, China) at a final concentration of 0.1-0.5 μg μl^-1^ and then printed on amino-silaned glass slides with a SmartArrayer^TM^ microarrayer (CapitalBio Corp). Each clone was printed triplicate. The particular procedures for microarray fabrication were conducted according to [37].

The relative gene expression profiles of QS at four developmental stages (SF, MF, BF and OV) compared with the corresponding four stages of EG were investigated by microarray analysis. For each stage, three sets of total RNA samples were extracted independently, and then RNA pool was constructed by mixing aliquot of RNA from the three sets of RNA samples. An aliquot of 5 μg total RNA from the RNA pool was used to produce Cy5/Cy3-labelled cDNA employing an RNA amplification combined with Klenow enzyme labeling strategy according to the protocol by [78]. Cy5/Cy3-labelled cDNA was hybridized with the microarray at 42°C overnight. Hybridization was performed in duplicate by dye swap (Cy5-labelled cDNA of QS versus Cy3-labelled cDNA of EG, and Cy5-labelled cDNA of EG versus Cy3-labelled cDNA of QS). And then the arrays were washed with 0.2% SDS, 2 × SSC at 42°C for 5 min, and 0.2% SSC for 5 min at room temperature.

### Microarray data analysis and EST sequence analysis

Arrays were scanned with a confocal laser scanner, LuxScan^TM^-scanner (CapitalBio Corp.) and the resulting images were analyzed with LuxScan^TM^ 3.0 software (CapitalBio Corp.). cDNA spots were screened and identified with the methods described by [77]. A spatial and intensity-dependent (LOWESS) normalization method was employed and normalized ratio data were then log transformed [79]. Differentially expressed genes were identified using a t-test, and multiple test corrections were performed using FDR. Genes with FDR <0.05 and a fold change ≥2 were identified as differentially expressed genes.

All the clones differentially expressed in at least one of the four stages were subjected to single-pass sequence using standard high throughput sequencing by BGI-Wuhan, China. All sequences were edited to omit vectors and low quality segments at 5’ and 3’ ends, then removal of sequences shorter than 100 bp with SeqClean software. Sequence reads were assembled by CAP3 program [80] with default parameters. Then all the unigenes were annotated using BLASTx with a cut-off value of 1.0 × e^-5^ by searching the UniProt database ( http://www.ebi.ac.uk/uniprot/). GO-KEGG-EC annotation was performed based on Annot8r platform [81]. Hierarchical clustering of transcript accumulation was performed with Cluster software (version 3.0) [82].

### Quantitative real-time PCR verification and candidate TFs analysis

Total RNA was extracted from QS and EG collected at four different developmental stages with the Trizol methods mentioned above. Primer pairs were designed with the Primer Express software (Applied Biosystems, Foster City, CA, USA). Primer sequences of 11 candidate genes for verification were provided in Additional file 5: Table S1, and primer sequences of 10 TFs were provided in Additional file 6: Table S2. Single strand cDNA was synthesized with the prescription of the Revert Aid TM first strand cDNA synthesis Kit (Fermentas, Life Science, EU). Then each cDNA sample was pre-amplified using the citrus house-keeping gene β-actin and normalized for subsequent real-time quantitative PCR (qRT-PCR). The PCR program differed in terms of the annealing temperature of each primer pair and the length of the predicted PCR products. The qRT-PCR was performed using the ABI 7500 Real Time System (PE Applied Biosystems, Foster City, CA, USA) with the method as described by [83]. And relative transcript change was analyzed by 2^-ΔΔc(t)^.

## Abbreviations

AP2: APETALA2; AFLP: Amplified fragment length polymorphism; C2H2 TF: Cys2His2 transcription factor; CBF: C-repeat DNA replication-related element binding factor; DREB: DNA replication-related element binding; ERF/EREBPs: Ethylene-responsive element binding proteins; MSAP: Methylation-sensitive amplification polymorphism; NAC TFs: (NAM, ATAF and CUC) transcription factors; qRT-PCR: Quantitative reverse transcription polymerase chain reaction; SCAR: Sequence characterized amplified region; SSR: Simple sequence repeat.

## Competing interests

The authors declare that they have no competing interests.

## Authors' contribution

WMQ performed the experiments and interpreted the results. ADZ carried out the ESTs annotation. YW helped with phenotype analysis. WMQ and WWG drafted the manuscript. LJC and XXG participated in the SSH-cDNA construction. XXD participated in research design. WWG proposed and supervised the overall project. All authors read and approved the final manuscript.

## Supplementary Material

Additional file 1 **Figure S1.** Comparison of expression patterns of 4 ESTs assembled the some contig encoding Methionine synthase. Y-axis represents the average Cy5 (Cy3) to Cy3 (Cy5) ratio in array hybridization. X-axis represents the four developmental stages. Click here for file

Additional file 2 **Figure S2.** The correlation of gene expression ratios between cDNA microarray and qRT-PCR. Data were from 11 probe sets at four developmental stages. The gene expression ratios based on cDNA microarray were in log2 transformed. Click here for file

Additional file 3 Sequences shorter than 200 bp but longer than 100 bp. Click here for file

Additional file 4 **Figure S3.** The purified PCR products for microarray probe. 100 bp molecular ladders were used. Click here for file

Additional file 5 **Table S1.** qRT-PCR primers for 11 candidate genes and citrus actin gene. Click here for file

Additional file 6 **Table S2.** qRT-PCR primers for 10 transcription factors (TFs). Click here for file

## References

[B1] Varoquaux F Blanvillain R Delseny M Gallois P Less is better: new approaches for seedless fruit production Trends Biotechnol 2000 18 233 242 10.1016/S0167-7799(00)01448-7 10802558

[B2] Gillaspy G Ben-David H Gruissem W Fruits: A developmental perspective Plant Cell 1993 5 1439 1451 12271039 10.1105/tpc.5.10.1439 PMC160374

[B3] Weterings K Russell SD Experimental analysis of the fertilization process Plant Cell 2004 16 S107 S118 10.1105/tpc.016873 15010512 PMC2643384

[B4] Fos M Proano K Nuez F Garcia-Martinez JL Role of gibberellins in parthenocarpic fruit development induced by the genetic system pat-3/pat-4 in tomato Physiol Plant 2001 111 545 550 10.1034/j.1399-3054.2001.1110416.x 11299021

[B5] Dorcey E Urbez C Blazquez MA Carbonell J Perez-Amador MA Fertilization-dependent auxin response in ovules triggers fruit development through the modulation of gibberellin metabolism in Arabidopsis Plant J 2009 58 318 332 10.1111/j.1365-313X.2008.03781.x 19207215

[B6] Serrani JC Carrera E Ruiz-Rivero O Gallego-Giraldo L Peres LEP Garcia-Martinez JL Inhibition of Auxin Transport from the Ovary or from the Apical Shoot Induces Parthenocarpic Fruit-Set in Tomato Mediated by Gibberellins Plant Physiol 2010 153 851 862 10.1104/pp.110.155424 20388661 PMC2879769

[B7] Plackett AR Thomas SG Wilson ZA Hedden P Gibberellin control of stamen development: a fertile field Trends Plant Sci 2011 16 568 578 10.1016/j.tplants.2011.06.007 21824801

[B8] Song S Qi T Huang H Ren Q Wu D Chang C Peng W Liu Y Peng J Xie D The Jasmonate-ZIM domain proteins interact with the R2R3-MYB transcription factors MYB21 and MYB24 to affect Jasmonate-regulated stamen development in Arabidopsis Plant Cell 2011 23 1000 1013 10.1105/tpc.111.083089 21447791 PMC3082250

[B9] Talon M Gmitter FG Jr Citrus genomics Int J Plant Genomics 2008 2008 528361 18509486 10.1155/2008/528361 PMC2396216

[B10] Deng XX Advances on citrus breeding in the world Acta Hortic Sinica 2005 32 1140 1146

[B11] Garcia-Lor A Garcia-Martinez JL Perez-Amador MA Identification of ovule and seed genes from Citrus clementina Tree Genet Genomes 2011 8 227 235

[B12] Zheng TG Qiu WM Fan GE Zheng BB Guo WW Construction and characterization of a cDNA library from floral organs and fruitlets of *Citrus reticulata* Biol Plantarum 2011 55 431 436 10.1007/s10535-011-0107-6

[B13] Cai XD Fu J Deng XX Guo WW Production and molecular characterization of potential seedless cybrid plants between pollen sterile Satsuma mandarin and two seedy Citrus cultivars Plant Cell Tiss Org 2007 90 275 283 10.1007/s11240-007-9266-8

[B14] Fu J Peng ZJ Cai XD Guo WW Regeneration and molecular characterization of interspecific somatic hybrids between Satsuma mandarin and two seedy sweet oranges for scion improvement Plant Breeding 2011 130 287 290 10.1111/j.1439-0523.2010.01773.x

[B15] Grosser JW Gmitter FGJ Protoplast fusion for production of tetraploids and triploids: applications for scion and rootstock breeding in citrus Plant Cell Tiss Org 2011 104 343 357 10.1007/s11240-010-9823-4

[B16] Guo WW Prasad D Cheng YJ Serrano P Deng XX Grosser JW Targeted cybridization in citrus: transfer of Satsuma cytoplasm to seedy cultivars for potential seedlessness Plant Cell Rep 2004 22 752 758 10.1007/s00299-003-0747-x 14730385

[B17] Vardi A Levin I Carmi N Induction of seedlessness in citrus: From classical techniques to emerging biotechnological approaches J Am Soc Hortic Sci 2008 133 117 126

[B18] Sun K Hunt K Hauser BA Ovule abortion in Arabidopsis triggered by stress Plant Physiol 2004 135 2358 2367 10.1104/pp.104.043091 15299130 PMC520803

[B19] Mezzetti B Landi L Pandolfini T Spena A The defH9-iaaM auxin-synthesizing gene increases plant fecundity and fruit production in strawberry and raspberry Biotechnol 2004 4 4 10.1186/1472-6750-4-4 PMC394336 15113427

[B20] Costantini E Landi L Silvestroni O Pandolfini T Spena A Mezzetti B Auxin synthesis-encoding transgene enhances grape fecundity Plant Physiol 2007 143 1689 1694 10.1104/pp.106.095232 17337528 PMC1851826

[B21] Hartley RW Barnase and barstar. Expression of its cloned inhibitor permits expression of a cloned ribonuclease J Mol Biol 1988 202 913 915 10.1016/0022-2836(88)90568-2 3050134

[B22] Li DD Shi W Deng XX Agrobacterium-mediated transformation of embryogenic calluses of Ponkan mandarin and the regeneration of plants containing the chimeric ribonuclease gene Plant Cell Rep 2002 21 153 156 10.1007/s00299-002-0492-6

[B23] Hanania U Velcheva M Or E Flaishman M Sahar N Perl A Silencing of chaperonin 21, that was differentially expressed in inflorescence of seedless and seeded grapes, promoted seed abortion in tobacco and tomato fruits Transgenic Res 2007 16 515 525 10.1007/s11248-006-9044-0 17103240

[B24] Hanania U Velcheva M Sahar N Flaishman M Or E Degani O Perl A The ubiquitin extension protein S27a is differentially expressed in developing flower organs of Thompson seedless versus Thompson seeded grape isogenic clones Plant Cell Rep 2009 28 1033 1042 10.1007/s00299-009-0715-1 19479258

[B25] Tan B Li DL Xu SX Fan GE Fan J Guo WW Highly efficient transformation of the GFP and MAC12.2 genes into precocious trifoliate orange (Poncirus trifoliata [L.] Raf), a potential model genotype for functional genomics studies in Citrus Tree Genet Genomes 2009 5 529 537 10.1007/s11295-009-0206-0

[B26] Li X Gao X Wei Y Deng L Ouyang Y Chen G Zhang Q Wu C Rice APOPTOSIS INHIBITOR5 coupled with two DEAD-box adenosine 5'-triphosphate-dependent RNA helicases regulates tapetum degeneration Plant Cell 2011 23 1416 1434 10.1105/tpc.110.082636 21467577 PMC3101562

[B27] Zhang D Liang W Yin C Zong J Gu F OsC6, encoding a lipid transfer protein, is required for postmeiotic anther development in rice Plant Physiol 2010 154 149 162 10.1104/pp.110.158865 20610705 PMC2938136

[B28] Zhu J Chen H Li H Gao JF Jiang H Wang C Guan YF Yang ZN Defective in Tapetal development and function 1 is essential for anther development and tapetal function for microspore maturation in Arabidopsis Plant J 2008 55 266 277 10.1111/j.1365-313X.2008.03500.x 18397379

[B29] Dun X Zhou Z Xia S Wen J Yi B Shen J Ma C Tu J Fu T BnaC.Tic40, a plastid inner membrane translocon originating from Brassica oleracea, is essential for tapetal function and microspore development in Brassica napus Plant J 2011 68 532 545 10.1111/j.1365-313X.2011.04708.x 21756273

[B30] Tang S Hu B Selecting of a seedless, good-quality and high-yield cultivar of ponkan-Qianyang seedless Ponkan South China Fruits 2001 30 3 6

[B31] Xiao JP Tan JJ Liu HL Chen LG Ye WQ Cheng WL Studies on the seedless mechanism of Lipeng No. 2 Ponkan (Citrus reticulata) Journal of Fruit Science 2007 24 421 426

[B32] Diatchenko L Lau YF Campbell AP Chenchik A Moqadam F Huang B Lukyanov S Lukyanov K Gurskaya N Sverdlov ED Siebert PD Suppression subtractive hybridization: a method for generating differentially regulated or tissue-specific cDNA probes and libraries Proc Natl Acad Sci U S A 1996 93 6025 6030 10.1073/pnas.93.12.6025 8650213 PMC39182

[B33] International Citrus Genome Consortium Haploid Clementine Genome 2011 http://int-citrusgenomics.org, http://www.phytozome.net/

[B34] Gmitter FG Jr Chen CX Machado MA de Souza AA Ollitrault P Froehlicher Y Shimizu T Citrus genomics Tree Genet Genomes 2012 8 611 626 10.1007/s11295-012-0499-2

[B35] Coetzer N Gazendam I Oelofse D Berger DK SSHscreen and SSHdb, generic software for microarray based gene discovery: application to the stress response in cowpea Plant Methods 2010 6 10 10.1186/1746-4811-6-10 20359330 PMC2859861

[B36] Hillmann A Dunne E Kenny D cDNA amplification by SMART-PCR and suppression subtractive hybridization (SSH)-PCR Methods Mol Biol 2009 496 223 243 10.1007/978-1-59745-553-4_15 18839114

[B37] Liu Q Zhu A Chai L Zhou W Yu K Ding J Xu J Deng X Transcriptome analysis of a spontaneous mutant in sweet orange [Citrus sinensis (L.) Osbeck] during fruit development J Exp Bot 2009 60 801 813 10.1093/jxb/ern329 19218315 PMC2652045

[B38] Wu ZD Soliman KM Bolton JJ Saba S Jenkins JN Identification of differentially expressed genes associated with cotton fiber development in a chromosomal substitution line (CS-B22sh) Funct Integr Genomic 2008 8 165 174 10.1007/s10142-007-0064-5 18043952

[B39] Gou X Yuan T Wei X Russell SD Gene expression in the dimorphic sperm cells of Plumbago zeylanica: transcript profiling, diversity, and relationship to cell type Plant J 2009 60 33 47 10.1111/j.1365-313X.2009.03934.x 19500307

[B40] Breto MP Ruiz C Pina JA Asins MJ The diversification of Citrus clementina Hort. ex Tan., a vegetatively propagated crop species Mol Phylogenet Evol 2001 21 285 293 10.1006/mpev.2001.1008 11697922

[B41] Butelli E Licciardello C Zhang Y Liu J Mackay S Bailey P Reforgiato-Recupero G Martin C Retrotransposons control fruit-specific, cold-dependent accumulation of anthocyanins in blood oranges Plant Cell 2012 24 1242 1255 10.1105/tpc.111.095232 22427337 PMC3336134

[B42] Xiao JP Chen LG Xie M Liu HL Ye WQ Identification of AFLP fragments linked to seedlessness in Ponkan mandarin (Citrus reticulata Blanco) and conversion to SCAR markers Sci Hortic-Amsterdam 2009 121 505 510 10.1016/j.scienta.2009.03.006

[B43] Scott RJ Spielman M Dickinson HG Stamen structure and function Plant Cell 2004 16 Suppl S46 60 10.1105/tpc.017012 15131249 PMC2643399

[B44] Wu G Amino acids: metabolism, functions, and nutrition Amino Acids 2009 37 1 17 19301095 10.1007/s00726-009-0269-0

[B45] Forde BG Lea PJ Glutamate in plants: metabolism, regulation, and signalling J Exp Bot 2007 58 2339 2358 10.1093/jxb/erm121 17578865

[B46] Kusano M Tabuchi M Fukushima A Funayama K Diaz C Kobayashi M Hayashi N Tsuchiya YN Takahashi H Kamata A Metabolomics data reveal a crucial role of cytosolic glutamine synthetase 1;1 in coordinating metabolic balance in rice Plant J 2011 66 456 466 10.1111/j.1365-313X.2011.04506.x 21255162

[B47] Gray BN Yang H Ahner BA Hanson MR An efficient downstream box fusion allows high-level accumulation of active bacterial beta-glucosidase in tobacco chloroplasts Plant Mol Biol 2011 76 345 355 10.1007/s11103-011-9743-7 21279422

[B48] Brzobohaty B Moore I Kristoffersen P Bako L Campos N Schell J Palme K Release of active cytokinin by a beta-glucosidase localized to the maize root meristem Science 1993 262 1051 1054 10.1126/science.8235622 8235622

[B49] Gerardi C Blando F Santino A Zacheo G Purification and characterisation of a beta-glucosidase abundantly expressed in ripe sweet cherry (Prunus avium L.) fruit Plant Sci 2001 160 795 805 10.1016/S0168-9452(00)00423-4 11297776

[B50] Thorlby G Fourrier N Warren G The SENSITIVE TO FREEZING2 gene, required for freezing tolerance in Arabidopsis thaliana, encodes a beta-glucosidase Plant Cell 2004 16 2192 2203 10.1105/tpc.104.024018 15258268 PMC519207

[B51] Munoz-Amatriain M Svensson JT Castillo AM Close TJ Valles MP Microspore embryogenesis: assignment of genes to embryo formation and green vs. albino plant production Funct Integr Genomics 2009 9 311 323 10.1007/s10142-009-0113-3 19229567 PMC2700865

[B52] Lam HM Peng SS Coruzzi GM Metabolic regulation of the gene encoding glutamine-dependent asparagine synthetase in Arabidopsis thaliana Plant Physiol 1994 106 1347 1357 10.1104/pp.106.4.1347 7846154 PMC159672

[B53] Olea F Perez-Garcia A Canton FR Rivera ME Canas R Avila C Cazorla FM Canovas FM de Vicente A Up-regulation and localization of asparagine synthetase in tomato leaves infected by the bacterial pathogen Pseudomonas syringae Plant Cell Physiol 2004 45 770 780 10.1093/pcp/pch092 15215512

[B54] Hwang IS An SH Hwang BK Pepper asparagine synthetase 1 (CaAS1) is required for plant nitrogen assimilation and defense responses to microbial pathogens Plant J 2011 67 749 762 10.1111/j.1365-313X.2011.04622.x 21535260

[B55] Maaroufi-Dguimi H Debouba M Gaufichon L Clement G Gouia H Hajjaji A Suzuki A An Arabidopsis mutant disrupted in ASN2 encoding asparagine synthetase 2 exhibits low salt stress tolerance Plant Physiol Biochem 2011 49 623 628 10.1016/j.plaphy.2011.03.010 21478030

[B56] Yang SF Hoffman NE Ethylene Biosynthesis and its Regulation in Higher Plants Annu Rev Plant Physiol 1984 35 155 189 10.1146/annurev.pp.35.060184.001103

[B57] Son GH Wan J Kim HJ Nguyen XC Chung WS Hong JC Stacey G Ethylene-responsive element-binding factor 5, ERF5, is involved in chitin-induced innate immunity response Mol Plant Microbe Interact 2012 25 48 60 10.1094/MPMI-06-11-0165 21936663

[B58] Moffat CS Ingle RA Wathugala DL Saunders NJ Knight H Knight MR ERF5 and ERF6 Play Redundant Roles as Positive Regulators of JA/Et-Mediated Defense against Botrytis cinerea in Arabidopsis PLoS One 2012 7 e35995 10.1371/journal.pone.0035995 22563431 PMC3338558

[B59] Pan Y Seymour GB Lu C Hu Z Chen X Chen G An ethylene response factor (ERF5) promoting adaptation to drought and salt tolerance in tomato Plant Cell Rep 2012 31 349 360 10.1007/s00299-011-1170-3 22038370

[B60] Bethke G Unthan T Uhrig JF Poschl Y Gust AA Scheel D Lee J Flg22 regulates the release of an ethylene response factor substrate from MAP kinase 6 in Arabidopsis thaliana via ethylene signaling Proc Natl Acad Sci U S A 2009 106 8067 8072 10.1073/pnas.0810206106 19416906 PMC2683104

[B61] Haake V Cook D Riechmann JL Pineda O Thomashow MF Zhang JZ Transcription factor CBF4 is a regulator of drought adaptation in Arabidopsis Plant Physiol 2002 130 639 648 10.1104/pp.006478 12376631 PMC166593

[B62] Xiao H Tattersall EA Siddiqua MK Cramer GR Nassuth A CBF4 is a unique member of the CBF transcription factor family of Vitis vinifera and Vitis riparia Plant Cell Environ 2008 31 1 10 17971068 10.1111/j.1365-3040.2007.01741.x

[B63] Lin RC Park HJ Wang HY Role of Arabidopsis RAP2.4 in regulating light- and ethylene-mediated developmental processes and drought stress tolerance Mol Plant 2008 1 42 57 10.1093/mp/ssm004 20031913

[B64] Krishnaswamy S Verma S Rahman MH Kav NN Functional characterization of four APETALA2-family genes (RAP2.6, RAP2.6L, DREB19 and DREB26) in Arabidopsis Plant Mol Biol 2011 75 107 127 10.1007/s11103-010-9711-7 21069430

[B65] Chandler JW Cole M Flier A Grewe B Werr W The AP2 transcription factors DORNROSCHEN and DORNROSCHEN-LIKE redundantly control Arabidopsis embryo patterning via interaction with PHAVOLUTA Development 2007 134 1653 1662 10.1242/dev.001016 17376809

[B66] Nag A Yang Y Jack T DORNROSCHEN-LIKE, an AP2 gene, is necessary for stamen emergence in Arabidopsis Plant Mol Biol 2007 65 219 232 10.1007/s11103-007-9210-7 17682829

[B67] Yang C Xu Z Song J Conner K Vizcay Barrena G Wilson ZA Arabidopsis MYB26/MALE STERILE35 regulates secondary thickening in the endothecium and is essential for anther dehiscence Plant Cell 2007 19 534 548 10.1105/tpc.106.046391 17329564 PMC1867336

[B68] Millar AA Gubler F The Arabidopsis GAMYB-like genes, MYB33 and MYB65, are MicroRNA-regulated genes that redundantly facilitate anther development Plant Cell 2005 17 705 721 10.1105/tpc.104.027920 15722475 PMC1069693

[B69] Higginson T Li SF Parish RW AtMYB103 regulates tapetum and trichome development in Arabidopsis thaliana Plant J 2003 35 177 192 10.1046/j.1365-313X.2003.01791.x 12848824

[B70] Yang ZN Zhang ZB Zhu J Gao JF Wang C Li H Li H Zhang HQ Zhang S Wang DM Transcription factor AtMYB103 is required for anther development by regulating tapetum development, callose dissolution and exine formation in Arabidopsis Plant J 2007 52 528 538 10.1111/j.1365-313X.2007.03254.x 17727613

[B71] Brownfield L Hafidh S Borg M Sidorova A Mori T Twell D A plant germline-specific integrator of sperm specification and cell cycle progression PLoS Genet 2009 5 e1000430 10.1371/journal.pgen.1000430 19300502 PMC2653642

[B72] Borg M Brownfield L Khatab H Sidorova A Lingaya M Twell D The R2R3 MYB Transcription Factor DUO1 Activates a Male Germline-Specific Regulon Essential for Sperm Cell Differentiation in Arabidopsis Plant Cell 2011 23 534 549 10.1105/tpc.110.081059 21285328 PMC3077786

[B73] Jung C Seo JS Han SW Koo YJ Kim CH Song SI Nahm BH Choi YD Cheong JJ Overexpression of AtMYB44 enhances stomatal closure to confer abiotic stress tolerance in transgenic Arabidopsis Plant Physiol 2008 146 623 635 18162593 10.1104/pp.107.110981 PMC2245844

[B74] Kirik V Kolle K Misera S Baumlein H Two novel MYB homologues with changed expression in late embryogenesis-defective Arabidopsis mutants Plant Mol Biol 1998 37 819 827 10.1023/A:1006011002499 9678577

[B75] Christianson JA Wilson IW Llewellyn DJ Dennis ES The low-oxygen-induced NAC domain transcription factor ANAC102 affects viability of Arabidopsis seeds following low-oxygen treatment Plant Physiol 2009 149 1724 1738 10.1104/pp.108.131912 19176720 PMC2663757

[B76] Liu YZ Liu Q Tao NG Deng XX Efficient isolation of RNA from fruit peel and pulp of ripening navel orange (Citrus sinensis Osbeck) Journal of Huazhong Agricultural University 2006 25 300 304

[B77] Ouyang B Yang T Li HX Zhang L Zhang YY Zhang JH Fei ZJ Ye ZB Identification of early salt stress response genes in tomato root by suppression subtractive hybridization and microarray analysis J Exp Bot 2007 58 507 520 17210988 10.1093/jxb/erl258

[B78] Guo Y Guo HY Zhang L Xie HY Zhao X Wang FX Li Z Wang YH Ma SL Tao JP Genomic analysis of anti-Hepatitis B virus (HBV) activity by small interfering RNA and lamivudine in stable HBV-producing cells J Virol 2005 79 14392 14403 10.1128/JVI.79.22.14392-14403.2005 16254373 PMC1280207

[B79] Yang YH Dudoit S Luu P Lin DM Peng V Ngai J Speed TP Normalization for cDNA microarray data: a robust composite method addressing single and multiple slide systematic variation Nucleic Acids Res 2002 30 e15 10.1093/nar/30.4.e15 11842121 PMC100354

[B80] Huang X Madan A CAP3: A DNA sequence assembly program Genome Res 1999 9 868 877 10.1101/gr.9.9.868 10508846 PMC310812

[B81] Schmid R Blaxter ML annot8r: GO, EC and KEGG annotation of EST datasets BMC Bioinforma 2008 9 180 10.1186/1471-2105-9-180 PMC2324097 18400082

[B82] Eisen MB Spellman PT Brown PO Botstein D Cluster analysis and display of genome-wide expression patterns Proc Natl Acad Sci U S A 1998 95 14863 14868 10.1073/pnas.95.25.14863 9843981 PMC24541

[B83] Liu Q Xu J Liu YZ Zhao XL Deng XX Guo LL Gu JQ A novel bud mutation that confers abnormal patterns of lycopene accumulation in sweet orange fruit (Citrus sinensis L. Osbeck) J Exp Bot 2007 58 4161 4171 10.1093/jxb/erm273 18182424

