# Supplementary figures and images for: Comparative transcript profiling of gene expression between seedless Ponkan mandarin and its seedy wild type during floral organ development by suppression subtractive hybridization and cDNA microarray
Source: BMC Genomics. 2012 Aug 16;13:397. doi: 10.1186/1471-2164-13-397 (PMC3495689; doi:10.1186/1471-2164-13-397)

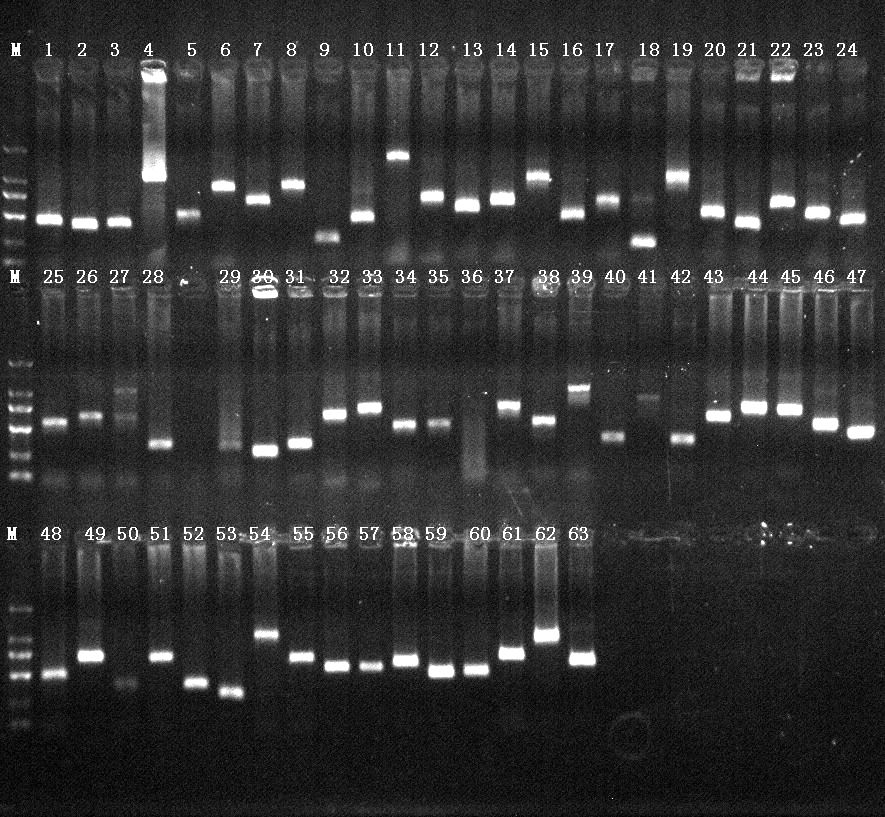


Figure 3S

Supplement: Additional file 3 — Sequences shorter than 200 bp but longer than 100 bp. [file 1471-2164-13-397-S3.doc]
