# Supplementary material for: Comparative transcript profiling of gene expression between seedless Ponkan mandarin and its seedy wild type during floral organ development by suppression subtractive hybridization and cDNA microarray
Source: BMC Genomics. 2012 Aug 16;13:397. doi: 10.1186/1471-2164-13-397 (PMC3495689; doi:10.1186/1471-2164-13-397)
Supplement: Additional file 4 — Figure S3. The purified PCR products for microarray probe. 100 bp molecular ladders were used. [file 1471-2164-13-397-S4.doc]

Table S1. List of qRT-PCR primers for 11 candidate genes and citrus actin gene.

| **Gene detected** | **Primer** |  | **Annealing**  **temperature (℃)** | **Amplicon**  **size (bp)** |
| --- | --- | --- | --- | --- |
|  | **Name** | **Sequence (5’ to 3’)** |  |  |
| JU497309 | F  R | TCCGTACCATTTTGCCCTTT  GAAGACGAAGTCACATCAATCATGA | 56  58 | 60 |
| JU497321 | F  R | CACGGCGGACTCTCCTAAGT  CCCAACCACCACAATTGGA | 62  58 | 59 |
| JU497323 | F  R | CACGGGACCCTTGATTGG  TGGTCCCACTTCCTGGAATCT | 60  60 | 60 |
| JU497332 | F  R | ATGCCGAGACTGCTTGTGAA  TAGTGGCGGTGGTGAAAGAAG | 58  60 | 57 |
| JU497344 | F  R | GATCACTGGAGTCGACCCTTCT  GGCGGTCCCAGTCATGTC | 62  62 | 60 |
| JU497351 | F  R | GCCGATCAGCCAAGTTTAATTT  GCGCCCGCCTGATATGT | 58  59 | 56 |
| JU497354 | F  R | AGCTCGTTGCGATGAAGAAAA  CGCTTGGCATCCATGTGAT | 56  58 | 58 |
| JU497370 | F  R | TCGACATGAAAGTCCGTGGAT  CCTGGGTCGAGCTGATGAA | 58  60 | 59 |
| JU497378 | F  R | TTCAGCTCTCCTTTTGGCTTCT  TCACCGAGCGAGCTCATG | 58  60 | 59 |
| JU497389 | F  R | CGAACTGGCAGTGGGAAGA  CCGGCTGGCTCGACTAGAC | 60  64 | 62 |
| JU497405 | F  R | GCCCCTTTTGGGCTAGAATC  GGACCACTCGACGGCTACTG | 60  64 | 58 |
| Actin | F  R | CCAAGCAGCATGAAGATGAA  ATCTGCTGGAAGGTGCTGAG | 60  60 | 100 |
