# Supplementary material for: Comparative transcript profiling of gene expression between seedless Ponkan mandarin and its seedy wild type during floral organ development by suppression subtractive hybridization and cDNA microarray
Source: BMC Genomics. 2012 Aug 16;13:397. doi: 10.1186/1471-2164-13-397 (PMC3495689; doi:10.1186/1471-2164-13-397)
Supplement: Additional file 5 — Table S1. qRT-PCR primers for 11 candidate genes and citrus actin gene. [file 1471-2164-13-397-S5.doc]

Table S2. List of quantitative reverse transcription PCR primers for 10 TFs

| **Gene detected** | **Primer** |  | **Annealing**  **temperature (℃)** | **Amplicon**  **size (bp)** |
| --- | --- | --- | --- | --- |
|  | **Name** | **Sequence (5’ to 3’)** |  |  |
| AP2-EREBP TF1 | F  R | GATCCAATAAAGACCCGTCAAAA  TTTCTGAGGCAACGGTTCAGT | 57  58 | 60 |
| AP2-EREBP TF2 | F  R | GTCCTGGCGTCGGTGTTAA  AGACCCAGCTGACCCATCAC | 60  62 | 56 |
| AP2-EREBP TF3 | F  R | TCCGGTTGGTGAAATGATGA  AAACCGTCGCTGAAAATCTCA | 56  56 | 56 |
| AP2/ERF-domain containing TF1 | F  R | GCCGATCAGCCAAGTTTAATTT  GCGCCCGCCTGATATGT | 58  59 | 56 |
| AP2/ERF-domain containing TF2 | F  R | AACTGGCCAAAGCAAGGAGAT  TGGGATGATGGAAATGGGATA | 58  56 | 59 |
| CBF/DREB-like protein | F  R | ATGCCGAGACTGCTTGTGAA  TAGTGGCGGTGGTGAAAGAAG | 58  60 | 57 |
| GATA TF | F  R | GGCCAAAAACCCTTTGCAAT  GGGAAGAGCCTGCCAGACTT | 56  62 | 58 |
| C2H2 type Zinc finger | F  R | TTGGGCGGACACAAACG  TGACGTCACTCCGCTCTTCTC | 57  57 | 57 |
| R2r3-MYB TF | F  R | CCGGGTCCGAAAATGGAT  TGTGTTGATGCCACCTGGTT | 57  58 | 58 |
| NAC domain protein | F  R | CATGAATATCGCCCTGCTAATG  CGTCAAGCCTTAGGTTGTTTTTG | 58  58 | 64 |
