# Supplementary material for: Comparative transcript profiling of gene expression between seedless Ponkan mandarin and its seedy wild type during floral organ development by suppression subtractive hybridization and cDNA microarray
Source: BMC Genomics. 2012 Aug 16;13:397. doi: 10.1186/1471-2164-13-397 (PMC3495689; doi:10.1186/1471-2164-13-397)
Supplement: Additional file 6 — Table S2. qRT-PCR primers for 10 transcription factors (TFs). [file 1471-2164-13-397-S6.doc]

>F2-N3

ACTTACAATCTCTTCTTCAACTACAAGCAAGCCAAAATCTCCCCAGAGACAATTGGCGCCACTTGCGACGGCGATGCCGGCAGCCGCGACGAGAAGTTGACACGCAACTCATCGGCGTCGTCTCTCCTCATCTCGTCTTCGAAATTC

>F10-E24

ATCTCAGATACGTCAGGCCCCGCTGCGCAGATTACACCTTACTGAATTAGCGAGCAGGACGAAGAGGCCTACAGGTTTAAGGAACGTTACTGCCTGCTGGTTCACGTCCAATTAATACGGCTGTATTTATTATTTCCTATTAATAAGTGTGATCTGGTTAAAAATAATGTTATGACCTAATAATATAGTAT

>R1-B15

AACATAAGATAAACCGATAATAGGAATCAGACAGATATCACTTTTTCTTGGCCAAGAACAGGCCCCATCGCTGCTCACCAGCTGAAGTCCTGATCAGCTTTGCCTTCCATCCGCCAACAATGTCATTGTAGTCTTCAT

>R2-N6

ATCCCCCCTCCTTTTTTTTTTTTTTTTTTCTTTTTGAGAATGATTAAACCTTAATAAGAATGATCAATGATATTCTTAGTCCAACAATTGCTTTGTCAAACATTGAATCAATTTAAGACAAAACATCGCTTAACAATCCTTGAGAAACAGAAATTCGCTACAGGCATCACAGATGAAACAAAATCAGCATGGTAT

>R5-N17

ATACAAGCTTTTTTTTTTTTTTTTTTTTTTTTTTTGGTAAACCCATTGGGACAGTCGTTCATTGAATACTAAATTTTACACACAGGGCCATACGCAGAATATCTCCAGCTTCGTTTTATTAGGGT
